# (2-Amino-7-methyl-4-oxidopteridine-6-carboxyl­ato-κ^3^ *O* ^4^,*N* ^5^,*O* ^6^)(ethane-1,2-diamine-κ^2^ *N*,*N*′)(1*H*-imidazole-κ*N* ^3^)nickel(II) dihydrate

**DOI:** 10.1107/S1600536813005898

**Published:** 2013-03-09

**Authors:** Siddhartha S. Baisya, Parag S. Roy

**Affiliations:** a Department of Chemistry, University of North Bengal, Siliguri 734 013, India

## Abstract

In the title complex, [Ni(C_8_H_5_N_5_O_3_)(C_2_H_8_N_2_)(C_3_N_2_H_4_)]·2H_2_O, a tridentate 2-amino-7-methyl-4-oxidopteridine-6-carboxyl­ate (pterin) ligand, a bidentate ancillary ethane-1,2-diamine (en) ligand and a monodentate 1*H*-imidazole (im) ligand complete a distorted octa­hedral geometry around the Ni^II^ atom. The pterin ligand forms two chelate rings. Both the en and im ligands are arranged nearly orthogonally relative to the pterin ligand [dihedral angles between the mean planes of the en and pterin ligands and of the im and pterin ligands are 84.62 (9) and 85.14 (9)°, respectively]. N—H⋯N, N—H⋯O, O—H⋯N and O—H⋯O hydrogen bonds link the complex mol­ecules and lattice water mol­ecules into a three-dimensional network.

## Related literature

For the importance of pterin in metalloenzymes, see: Basu & Burgmayer (2011 ▶); Burgmayer (1998 ▶); Fitzpatrick (2003 ▶); Fukuzumi & Kojima (2008 ▶); Kaim *et al.* (1999 ▶). For the structures of related nickel complexes, see: Baisya & Roy (2013 ▶); Crispini *et al.* (2005 ▶). For the structures of related copper complexes, see: Odani *et al.* (1992 ▶). For the electron-shuffling ability of the pterin unit as well as its donor groups and the effect on the geometric parameters of related complexes, see: Beddoes *et al.* (1993 ▶); Kohzuma *et al.* (1988 ▶); Russell *et al.* (1992 ▶). For the synthesis of the pterin ligand, see: Wittle *et al.* (1947 ▶). For refinement of H atoms, see: Cooper *et al.* (2010 ▶).
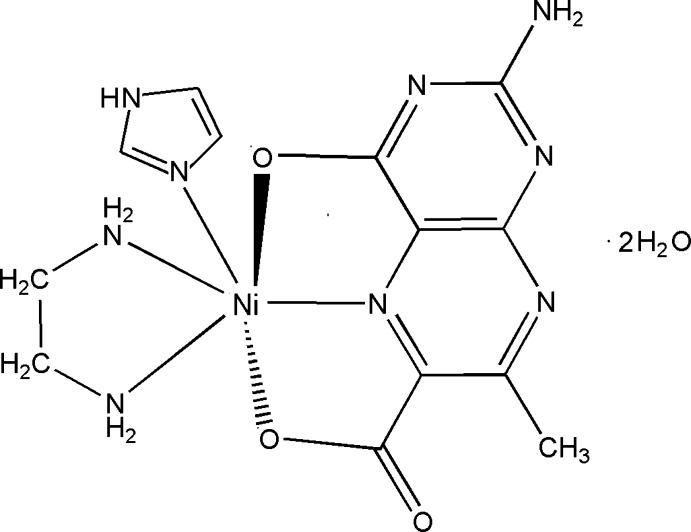


## Experimental

### 

#### Crystal data

[Ni(C_8_H_5_N_5_O_3_)(C_2_H_8_N_2_)(C_3_H_4_N_2_)]·2H_2_O
*M* *_r_* = 442.08
Orthorhombic, 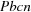

*a* = 13.484 (2) Å
*b* = 8.8741 (15) Å
*c* = 29.959 (5) Å
*V* = 3584.9 (10) Å^3^
*Z* = 8
Mo *K*α radiation
μ = 1.13 mm^−1^
*T* = 293 K
0.24 × 0.24 × 0.03 mm

#### Data collection

Bruker Kappa APEXII CCD diffractometer
Absorption correction: multi-scan (*SADABS*; Sheldrick, 1996 ▶) *T*_min_ = 0.77, *T*_max_ = 0.97
19640 measured reflections
4231 independent reflections
3521 reflections with *I* > 2σ(*I*)
*R*_int_ = 0.030

#### Refinement

*R*[*F* ^2^ > 2σ(*F* ^2^)] = 0.036
*wR*(*F* ^2^) = 0.091
*S* = 0.95
4231 reflections
253 parameters
H-atom parameters constrained
Δρ_max_ = 0.62 e Å^−3^
Δρ_min_ = −0.32 e Å^−3^

### 

Data collection: *APEX2* (Bruker, 2007 ▶); cell refinement: *SAINT* (Bruker, 2007 ▶); data reduction: *SAINT*; program(s) used to solve structure: *SHELXS97* (Sheldrick, 2008 ▶); program(s) used to refine structure: *CRYSTALS* (Betteridge *et al.*, 2003 ▶); molecular graphics: *CAMERON* (Watkin *et al.*, 1996 ▶); software used to prepare material for publication: *CRYSTALS*.

## Supplementary Material

Crystal structure: contains datablock(s) I, global. DOI: 10.1107/S1600536813005898/hy2617sup1.cif

Structure factors: contains datablock(s) I. DOI: 10.1107/S1600536813005898/hy2617Isup2.hkl

Additional supplementary materials:  crystallographic information; 3D view; checkCIF report

## Figures and Tables

**Table 1:** Hydrogen-bond geometry (Å, °)

*D*—H⋯*A*	*D*—H	H⋯*A*	*D*⋯*A*	*D*—H⋯*A*
N5—H171⋯O4^i^	0.84	2.50	3.193 (3)	140
N5—H172⋯N4^ii^	0.85	2.13	2.984 (3)	177
N6—H182⋯O5	0.88	2.28	3.099 (3)	154
N7—H211⋯N2^iii^	0.90	2.41	3.298 (2)	173
N7—H212⋯O4^iv^	0.87	2.53	3.210 (3)	136
N9—H241⋯O5^v^	0.89	2.15	3.024 (3)	168
O4—H271⋯N3^vi^	0.82	2.02	2.837 (2)	169
O4—H272⋯O3	0.81	2.07	2.859 (2)	167
O5—H281⋯O2^vii^	0.83	2.00	2.822 (2)	170
O5—H282⋯O1	0.81	2.14	2.789 (2)	138

Symmetry codes: (i) 
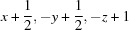
; (ii) 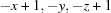; (iii) 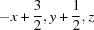; (iv) 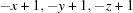; (v) 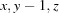; (vi) 
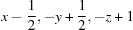
; (vii) 
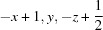
.

**Fig. 1.**
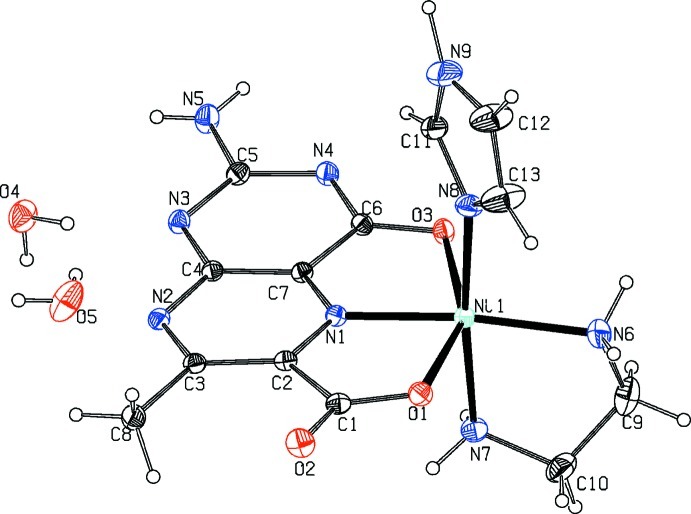
The molecular structure of the title compound. Displacement ellipsoids are drawn at the 30% probability level.

**Fig. 2.**
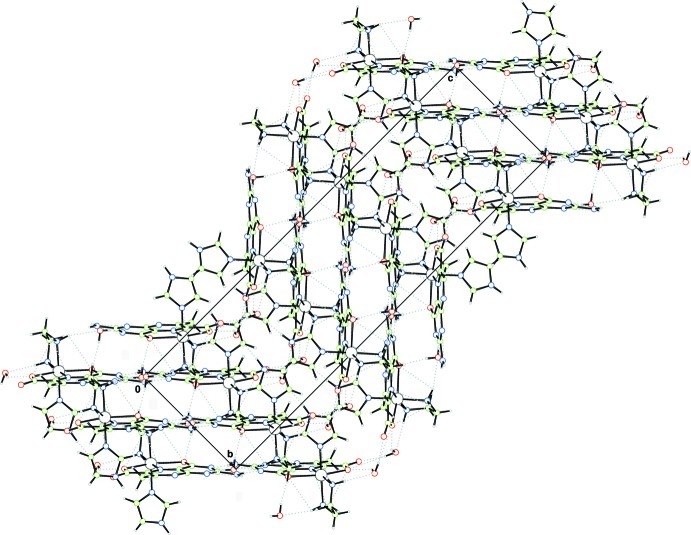
The crystal packing diagram of the title compound, viewed along the a axis. Dotted lines indicate hydrogen bonds.

## supplementary crystallographic information

### Comment

Increasing attention is being paid nowadays towards the metalloenzymes requiring both a pterin and a transition metal (Basu & Burgmayer, 2011; Burgmayer, 1998; Fitzpatrick, 2003; Fukuzumi & Kojima, 2008; Kaim *et al.*, 1999). This has, in turn, catalysed research work on the coordination chemistry of the bicyclic N-heterocycles called pteridines in general, and an important member of this family named pterin in particular. Literature survey reveals the existence of only a couple of structurally characterized Ni(II)–pterin complexes (Baisya & Roy, 2013; Crispini *et al.*, 2005) and no related quaternary complex. The present effort is concerned with the title quaternary complex, possessing a tridentate pterin ligand, a bidentate σ-donor ligand like en and a monodentate σ-donor ligand like im.

The molecular structure (Fig. 1) represents a mononuclear Ni^II^ centre in a distorted octahedral coordination geometry, with two N atoms (N6 and N7) of the en ligand, a pyrazine ring N atom (N1) of the pterin ligand and an imidazole ring N atom (N8), forming the equatorial plane. The two pterin O atoms (O1 and O3) occupy the longer axial positions, with the phenolate O3 constituting the longest axial bond [2.2722 (14) Å]. The pterin ligand forms two five-membered chelate rings having small bite angles [75.91 (6) and 77.50 (6)°], instead of only one per pterin ligand for an earlier case (Crispini *et al.*, 2005). This factor is responsible to a large extent for the observed distortion here from regular octahedral geometry. Accordingly, the O1—Ni1—O3 axis shows maximum deviation [153.37 (5)°] from linearity. Again, closest approach to linearity [174.10 (7)°] is observed for the N7—Ni1—N8 axis, which is associated with both the im and en ligands. Here each such ligand tries to achieve near orthogonality with respect to the pterin ligand [dihedral angles between the mean planes of the en and pterin ligands and of the im and pterin ligands are 84.62 (9) and 85.14 (9)°, respectively], for minimizing the steric repulsion. In line with the earlier observations on related copper complexes (Odani *et al.*, 1992), the pyrazine ring N atom (N1) is located in the equatorial plane. The corresponding short Ni1—N1 distance [1.9787 (16) Å] indicates dπ–pπ interaction between the pterin ring and the Ni^II^ atom (d^8^), with further assistance from the nearby π-donating phenolate and carboxylate O atoms (Kohzuma *et al.*, 1988). The pterin ligand is coordinated in its binegative form as an *O,N,O*-donor, as evident from the charge balance of this Ni(II) complex. The significantly shorter nature of the O3—C6 [1.271 (2) Å] and N5—C5 [1.332 (2) Å] bonds could be rationalized in terms of electron-shuffling ability of the pterin ring (Baisya & Roy, 2013; Beddoes *et al.*, 1993; Russell *et al.*, 1992).

In the crystal, intermolecular N—H···N, N—H···O, O—H···N and O—H···O hydrogen bonds (Table 1) link the complex molecules and lattice water molecules into a three-dimensional network (Fig 2). The lattice water molecules play a decisive role for the crystal packing.

### Experimental

2-Amino-4–hydroxy-7-methylpteridine-6-carboxylic acid sesquihydrate (C_8_H_7_N_5_O_3_.1.5H_2_O) was obtained by published procedure (Wittle *et al.*, 1947). The title complex was synthesized by the dropwise addition of an aqueous alkaline solution (NaOH: 11 mg, 0.275 mmol) of the pterin ligand (31 mg, 0.125 mmol) to a warm (311 K; paraffin oil bath) aqueous reaction mixture containing NiSO_4_.7H_2_O (35 mg, 0.125 mmol), ethane-1,2-diamine (7.5 mg, 0.125 mmol) and 1*H*-imidazole (14 mg, 0.2 mmol); final volume was 45 ml. The pH value was adjusted to 10.3 and the mixture was stirred for 3 h; final pH was 9.7. The orange coloured solution was transferred to a 100 ml beaker and allowed to stand at room temperature. Orange crystals appeared after 4 days (yield: 40%), which were suitable for single-crystal X-ray diffraction. Sample for analytical purpose could be obtained by filtration, repeated washing with small quantities of water and drying *in vacuo* over silica gel. Analysis, calculated for C_13_H_21_N_9_NiO_5_: C 35.31, H 4.80, N 28.52%; found: C 35.72, H 4.70, N 28.07%.

### Refinement

The H atoms were all located in a difference map, but those attached to C atoms were repositioned geometrically. The H atoms were initially refined with soft restrains on bond lengths and angles to regularize their geometry (C—H = 0.93–0.98, N—H = 0.86–0.89, O—H = 0.82 Å) and *U*_iso_(H) = 1.2–1.5*U*_eq_(parent atom), after which the positions were refined with rigiding constrains (Cooper *et al.*, 2010).

### Crystal data

**Table:**

[Ni(C_8_H_5_N_5_O_3_)(C_2_H_8_N_2_)(C_3_H_4_N_2_)]·2H_2_O	*F*(000) = 1840
*M**_r_* = 442.08	*D*_x_ = 1.638 Mg m^−^^3^
Orthorhombic, *P**b**c**n*	Mo *K*α radiation, λ = 0.71073 Å
Hall symbol: -P 2n 2ab	Cell parameters from 4231 reflections
*a* = 13.484 (2) Å	θ = 1.4–28.3°
*b* = 8.8741 (15) Å	µ = 1.13 mm^−^^1^
*c* = 29.959 (5) Å	*T* = 293 K
*V* = 3584.9 (10) Å^3^	Plate, orange
*Z* = 8	0.24 × 0.24 × 0.03 mm

### Data collection

**Table:**

Bruker Kappa APEXII CCD diffractometer	3521 reflections with *I* > 2σ(*I*)
Graphite monochromator	*R*_int_ = 0.030
φ and ω scans	θ_max_ = 28.3°, θ_min_ = 1.4°
Absorption correction: multi-scan (*SADABS*; Sheldrick, 1996)	*h* = −17→17
*T*_min_ = 0.77, *T*_max_ = 0.97	*k* = −11→11
19640 measured reflections	*l* = −24→38
4231 independent reflections	

### Refinement

**Table:**

Refinement on *F*^2^	Primary atom site location: structure-invariant direct methods
Least-squares matrix: full	Hydrogen site location: difference Fourier map
*R*[*F*^2^ > 2σ(*F*^2^)] = 0.036	H-atom parameters constrained
*wR*(*F*^2^) = 0.091	Method = Modified Sheldrick *w* = 1/[σ^2^(*F*^2^) + (0.04*P*)^2^ + 3.53*P*], where *P* = [max(*F*_o_^2^,0) + 2*F*_c_^2^]/3
*S* = 0.95	(Δ/σ)_max_ = 0.001
4231 reflections	Δρ_max_ = 0.62 e Å^−^^3^
253 parameters	Δρ_min_ = −0.32 e Å^−^^3^
0 restraints	

### Fractional atomic coordinates and isotropic or equivalent isotropic displacement parameters (Å^2^)

**Table:**

	*x*	*y*	*z*	*U*_iso_*/*U*_eq_	
Ni1	0.461327 (17)	0.49322 (3)	0.372395 (8)	0.0275	
O1	0.54402 (10)	0.60856 (16)	0.32134 (5)	0.0335	
C1	0.63525 (15)	0.5742 (2)	0.31846 (6)	0.0292	
O2	0.69402 (11)	0.62442 (18)	0.29021 (5)	0.0423	
C2	0.67092 (14)	0.4597 (2)	0.35277 (6)	0.0262	
N1	0.59680 (11)	0.40936 (17)	0.37761 (5)	0.0254	
C7	0.61252 (13)	0.3066 (2)	0.40867 (6)	0.0252	
C4	0.70658 (13)	0.2460 (2)	0.41611 (6)	0.0246	
N2	0.78491 (11)	0.29914 (18)	0.39231 (5)	0.0286	
C3	0.76772 (14)	0.4043 (2)	0.36124 (6)	0.0273	
C8	0.85670 (16)	0.4622 (2)	0.33676 (8)	0.0407	
H111	0.9180	0.4284	0.3494	0.0629*	
H113	0.8523	0.4298	0.3069	0.0630*	
H112	0.8565	0.5676	0.3363	0.0619*	
N3	0.71925 (12)	0.13478 (18)	0.44623 (5)	0.0293	
C5	0.63537 (14)	0.0864 (2)	0.46626 (6)	0.0297	
N4	0.54093 (12)	0.14309 (19)	0.46268 (5)	0.0312	
C6	0.52732 (13)	0.2575 (2)	0.43450 (6)	0.0268	
O3	0.44423 (10)	0.32158 (16)	0.42815 (5)	0.0329	
N5	0.64372 (14)	−0.0319 (2)	0.49338 (6)	0.0414	
H172	0.5918	−0.0634	0.5068	0.0504*	
H171	0.7003	−0.0668	0.4983	0.0503*	
N6	0.32580 (14)	0.6051 (2)	0.36971 (7)	0.0478	
C9	0.3190 (2)	0.7106 (3)	0.40720 (9)	0.0610	
C10	0.4178 (2)	0.7830 (3)	0.41328 (10)	0.0556	
N7	0.49397 (14)	0.66577 (19)	0.41780 (6)	0.0367	
H211	0.5548	0.7039	0.4136	0.0564*	
H212	0.4950	0.6335	0.4452	0.0559*	
H202	0.4167	0.8476	0.4384	0.0688*	
H201	0.4317	0.8402	0.3867	0.0695*	
H192	0.2669	0.7839	0.4035	0.0736*	
H191	0.3043	0.6540	0.4352	0.0746*	
H181	0.2742	0.5434	0.3674	0.0732*	
H182	0.3228	0.6546	0.3442	0.0727*	
N8	0.41434 (14)	0.3313 (2)	0.32768 (6)	0.0383	
C11	0.41880 (17)	0.1814 (2)	0.33182 (8)	0.0416	
N9	0.38881 (18)	0.1135 (3)	0.29413 (9)	0.0652	
C12	0.3599 (3)	0.2243 (3)	0.26639 (9)	0.0699	
C13	0.3786 (3)	0.3547 (3)	0.28659 (9)	0.0733	
H261	0.3671	0.4468	0.2743	0.0892*	
H251	0.3344	0.2134	0.2406	0.0848*	
H241	0.3806	0.0153	0.2892	0.0825*	
H231	0.4395	0.1296	0.3568	0.0527*	
O4	0.37876 (13)	0.4892 (2)	0.50415 (6)	0.0543	
H271	0.3379	0.4436	0.5195	0.0824*	
H272	0.3890	0.4340	0.4833	0.0825*	
O5	0.38933 (16)	0.7745 (2)	0.28388 (6)	0.0685	
H281	0.3645	0.7400	0.2605	0.1040*	
H282	0.4446	0.7401	0.2817	0.1049*	

### Atomic displacement parameters (Å^2^)

**Table:**

	*U*^11^	*U*^22^	*U*^33^	*U*^12^	*U*^13^	*U*^23^
Ni1	0.02605 (14)	0.02545 (14)	0.03107 (14)	0.00016 (9)	−0.00317 (9)	0.00076 (9)
O1	0.0339 (7)	0.0321 (7)	0.0344 (7)	−0.0011 (6)	−0.0042 (6)	0.0073 (6)
C1	0.0361 (10)	0.0262 (9)	0.0253 (9)	−0.0028 (8)	−0.0023 (7)	0.0001 (7)
O2	0.0456 (9)	0.0479 (9)	0.0334 (7)	−0.0033 (7)	0.0059 (6)	0.0132 (7)
C2	0.0310 (9)	0.0226 (8)	0.0250 (8)	−0.0023 (7)	0.0013 (7)	−0.0009 (7)
N1	0.0259 (7)	0.0252 (8)	0.0250 (7)	0.0000 (6)	−0.0009 (6)	0.0005 (6)
C7	0.0274 (9)	0.0237 (8)	0.0245 (8)	−0.0002 (7)	0.0010 (7)	−0.0007 (7)
C4	0.0271 (9)	0.0238 (8)	0.0228 (8)	−0.0001 (7)	0.0000 (7)	−0.0030 (7)
N2	0.0280 (8)	0.0257 (8)	0.0320 (8)	0.0009 (6)	0.0034 (6)	−0.0004 (6)
C3	0.0297 (10)	0.0236 (8)	0.0286 (9)	−0.0025 (7)	0.0039 (7)	−0.0027 (7)
C8	0.0323 (10)	0.0358 (11)	0.0541 (13)	0.0004 (9)	0.0119 (9)	0.0109 (10)
N3	0.0282 (8)	0.0304 (8)	0.0293 (8)	0.0033 (6)	0.0010 (6)	0.0046 (6)
C5	0.0340 (10)	0.0275 (9)	0.0275 (9)	0.0017 (8)	0.0013 (7)	0.0023 (7)
N4	0.0290 (8)	0.0321 (8)	0.0324 (8)	−0.0002 (6)	0.0042 (6)	0.0064 (7)
C6	0.0263 (9)	0.0281 (9)	0.0258 (8)	−0.0016 (7)	0.0007 (7)	−0.0003 (7)
O3	0.0255 (6)	0.0372 (7)	0.0359 (7)	0.0023 (6)	0.0030 (5)	0.0058 (6)
N5	0.0376 (10)	0.0412 (10)	0.0454 (10)	0.0058 (8)	0.0078 (8)	0.0188 (8)
N6	0.0336 (10)	0.0475 (11)	0.0624 (13)	0.0043 (8)	−0.0076 (9)	0.0034 (9)
C9	0.0523 (15)	0.0690 (18)	0.0617 (16)	0.0258 (14)	0.0095 (13)	0.0033 (14)
C10	0.0660 (17)	0.0399 (13)	0.0610 (16)	0.0132 (12)	−0.0007 (13)	−0.0132 (11)
N7	0.0413 (10)	0.0360 (9)	0.0329 (9)	−0.0008 (8)	−0.0013 (7)	−0.0027 (7)
N8	0.0422 (10)	0.0324 (9)	0.0402 (9)	−0.0059 (8)	−0.0084 (8)	−0.0020 (7)
C11	0.0428 (12)	0.0323 (11)	0.0498 (13)	−0.0009 (9)	−0.0073 (10)	−0.0025 (9)
N9	0.0756 (16)	0.0430 (12)	0.0769 (16)	−0.0070 (11)	−0.0090 (13)	−0.0200 (11)
C12	0.112 (3)	0.0476 (15)	0.0503 (15)	−0.0073 (16)	−0.0420 (16)	−0.0115 (12)
C13	0.122 (3)	0.0437 (14)	0.0544 (16)	−0.0051 (16)	−0.0438 (17)	0.0012 (12)
O4	0.0545 (10)	0.0558 (11)	0.0525 (10)	−0.0125 (8)	0.0175 (8)	−0.0034 (8)
O5	0.0879 (14)	0.0667 (12)	0.0509 (10)	0.0327 (11)	−0.0241 (10)	−0.0153 (9)

### Geometric parameters (Å, º)

**Table:**

Ni1—O1	2.1519 (14)	N5—H172	0.853
Ni1—N1	1.9787 (16)	N5—H171	0.837
Ni1—O3	2.2722 (14)	N6—C9	1.465 (3)
Ni1—N6	2.0812 (19)	N6—H181	0.888
Ni1—N7	2.0949 (17)	N6—H182	0.881
Ni1—N8	2.0638 (17)	C9—C10	1.491 (4)
O1—C1	1.270 (2)	C9—H192	0.964
C1—O2	1.242 (2)	C9—H191	0.997
C1—C2	1.523 (3)	C10—N7	1.468 (3)
C2—N1	1.324 (2)	C10—H202	0.945
C2—C3	1.418 (3)	C10—H201	0.962
N1—C7	1.320 (2)	N7—H211	0.896
C7—C4	1.395 (2)	N7—H212	0.869
C7—C6	1.452 (2)	N8—C11	1.338 (3)
C4—N2	1.359 (2)	N8—C13	1.338 (3)
C4—N3	1.348 (2)	C11—N9	1.342 (3)
N2—C3	1.338 (2)	C11—H231	0.922
C3—C8	1.497 (3)	N9—C12	1.346 (4)
C8—H111	0.957	N9—H241	0.890
C8—H113	0.940	C12—C13	1.329 (4)
C8—H112	0.936	C12—H251	0.850
N3—C5	1.351 (2)	C13—H261	0.910
C5—N4	1.373 (2)	O4—H271	0.825
C5—N5	1.332 (2)	O4—H272	0.807
N4—C6	1.333 (2)	O5—H281	0.834
C6—O3	1.271 (2)	O5—H282	0.808
			
O1—Ni1—N1	75.91 (6)	C7—C6—O3	118.92 (16)
O1—Ni1—O3	153.37 (5)	N4—C6—O3	123.85 (16)
N1—Ni1—O3	77.50 (6)	Ni1—O3—C6	108.69 (11)
O1—Ni1—N6	101.58 (7)	C5—N5—H172	118.4
N1—Ni1—N6	173.26 (7)	C5—N5—H171	118.4
O3—Ni1—N6	105.01 (7)	H172—N5—H171	122.9
O1—Ni1—N7	90.29 (6)	Ni1—N6—C9	109.29 (15)
N1—Ni1—N7	91.70 (7)	Ni1—N6—H181	113.4
O3—Ni1—N7	91.95 (6)	C9—N6—H181	113.8
N6—Ni1—N7	82.01 (8)	Ni1—N6—H182	108.1
O1—Ni1—N8	91.65 (7)	C9—N6—H182	110.0
N1—Ni1—N8	94.18 (7)	H181—N6—H182	101.8
O3—Ni1—N8	88.82 (7)	N6—C9—C10	108.2 (2)
N6—Ni1—N8	92.13 (8)	N6—C9—H192	112.9
N7—Ni1—N8	174.10 (7)	C10—C9—H192	112.0
Ni1—O1—C1	115.85 (12)	N6—C9—H191	109.6
O1—C1—O2	125.32 (18)	C10—C9—H191	107.0
O1—C1—C2	114.83 (16)	H192—C9—H191	107.0
O2—C1—C2	119.84 (17)	C9—C10—N7	109.3 (2)
C1—C2—N1	111.47 (16)	C9—C10—H202	110.1
C1—C2—C3	130.01 (16)	N7—C10—H202	111.5
N1—C2—C3	118.51 (16)	C9—C10—H201	107.5
C2—N1—Ni1	121.72 (13)	N7—C10—H201	108.3
C2—N1—C7	120.55 (16)	H202—C10—H201	110.0
Ni1—N1—C7	117.63 (12)	C10—N7—Ni1	108.13 (14)
N1—C7—C4	121.65 (16)	C10—N7—H211	111.1
N1—C7—C6	117.12 (16)	Ni1—N7—H211	112.2
C4—C7—C6	121.23 (16)	C10—N7—H212	109.4
C7—C4—N2	119.28 (16)	Ni1—N7—H212	112.0
C7—C4—N3	120.28 (16)	H211—N7—H212	104.0
N2—C4—N3	120.44 (16)	Ni1—N8—C11	128.20 (15)
C4—N2—C3	118.19 (16)	Ni1—N8—C13	126.88 (17)
C2—C3—N2	121.72 (16)	C11—N8—C13	104.8 (2)
C2—C3—C8	122.07 (17)	N8—C11—N9	110.8 (2)
N2—C3—C8	116.19 (17)	N8—C11—H231	125.8
C3—C8—H111	113.0	N9—C11—H231	123.4
C3—C8—H113	108.0	C11—N9—C12	106.2 (2)
H111—C8—H113	109.6	C11—N9—H241	128.0
C3—C8—H112	110.4	C12—N9—H241	125.3
H111—C8—H112	108.7	N9—C12—C13	107.5 (2)
H113—C8—H112	107.0	N9—C12—H251	126.4
C4—N3—C5	115.09 (15)	C13—C12—H251	126.1
N3—C5—N4	128.71 (17)	N8—C13—C12	110.6 (2)
N3—C5—N5	116.80 (17)	N8—C13—H261	124.9
N4—C5—N5	114.49 (17)	C12—C13—H261	124.4
C5—N4—C6	117.14 (16)	H271—O4—H272	104.5
C7—C6—N4	117.19 (16)	H281—O5—H282	99.5

### Hydrogen-bond geometry (Å, º)

**Table:**

*D*—H···*A*	*D*—H	H···*A*	*D*···*A*	*D*—H···*A*
N5—H171···O4^i^	0.84	2.50	3.193 (3)	140
N5—H172···N4^ii^	0.85	2.13	2.984 (3)	177
N6—H182···O5	0.88	2.28	3.099 (3)	154
N7—H211···N2^iii^	0.90	2.41	3.298 (2)	173
N7—H212···O4^iv^	0.87	2.53	3.210 (3)	136
N9—H241···O5^v^	0.89	2.15	3.024 (3)	168
O4—H271···N3^vi^	0.82	2.02	2.837 (2)	169
O4—H272···O3	0.81	2.07	2.859 (2)	167
O5—H281···O2^vii^	0.83	2.00	2.822 (2)	170
O5—H282···O1	0.81	2.14	2.789 (2)	138

Symmetry codes: (i) *x*+1/2, −*y*+1/2, −*z*+1; (ii) −*x*+1, −*y*, −*z*+1; (iii) −*x*+3/2, *y*+1/2, *z*; (iv) −*x*+1, −*y*+1, −*z*+1; (v) *x*, *y*−1, *z*; (vi) *x*−1/2, −*y*+1/2, −*z*+1; (vii) −*x*+1, *y*, −*z*+1/2.
